# Concurrent Acute Pyelonephritis in a Mother-Infant Dyad: A Case Report

**DOI:** 10.7759/cureus.112745

**Published:** 2026-07-15

**Authors:** Vivian Tran, Clement Lee

**Affiliations:** 1 College of Medicine, Drexel University, Philadelphia, USA; 2 School of Medicine, Tufts University, Boston, USA; 3 Departments of Medicine and Pediatrics, Newton-Wellesley Hospital, Newton, USA

**Keywords:** acute pyelo, infant microbiome, microbiome of the mother, uncomplicated urinary tract infection, uropathogenic escherichia coli

## Abstract

Acute pyelonephritis is an infection of the kidney that affects both adult and pediatric populations. We describe the case of a 31-year-old mother and her infant boy diagnosed with pyelonephritis due to the same organism. Both patients harbored risk factors that increased their susceptibility to infection; however, more importantly, maternal-to-neonatal transmission of microbiota is thought to play a large role in the neonate’s infection. This case highlights the importance of and the clinical implications of maternal and neonatal microbiomes.

## Introduction

Acute pyelonephritis is an infection of the kidney, often caused by ascending urinary tract infections (UTIs). Sexually active women are at high risk for UTIs [1]. However, neonates are also considered at high risk due to various risk factors, including underdeveloped immune systems and congenital genitourinary anomalies [2,3]. 

The gut microbiome of a pregnant woman plays a large role in establishing the gut microbiome of her children. Therefore, the link between the maternal microbiome and the neonatal microbiome is increasingly studied for its significance in child health outcomes. We present the case of a 31-year-old mother and her infant boy who were both hospitalized with pyelonephritis due to the same organism to discuss potential maternal-to-neonatal routes of microbiota transmission and their clinical implications. 

## Case presentation

A 31-year-old G1P1 woman brought her 13-day-old infant to the emergency department for poor feeding and fever. She noted that the baby had been fussier than usual, not feeding well, and had a weak cry. Rectal temperature was 100.5º F at home. Otherwise, the baby had been doing well. He was born via normal vaginal delivery at term gestation, and his birth admission was unremarkable. He had passed his newborn hearing and critical congenital heart disease screening and received prophylactic vitamin K, erythromycin eye ointment, and hepatitis B vaccination. Circumcision had been deferred per parental preference. Prenatal ultrasonography was normal, with no urinary tract dilation noted. He was breastfed and was last seen in his pediatrician’s office on the 10th day of life, with his weight at 95% of birth weight.

In the emergency department, a comprehensive septic workup was initiated. Cerebrospinal fluid (CSF) analysis was inconsistent with bacterial meningitis, blood cultures were negative, and urinalysis results are shown in Table 1; urine culture eventually grew >100,000 colony-forming units per mL of pan-susceptible Escherichia coli. He was diagnosed with neonatal pyelonephritis and treated with 14 days of antibiotics total (seven days of intravenous ampicillin followed by seven days of oral amoxicillin). 

**Table 1:** Urinalysis results of the infant

Laboratory Result	Result	Reference Value
White blood cells	>150/high-powered field (hpf)	0-5/hpf
Leukocyte esterase	3+	Negative
Nitrites	Positive	Negative
Red blood cells	50-100/hpf	0-3/hpf

**Figure 1:**
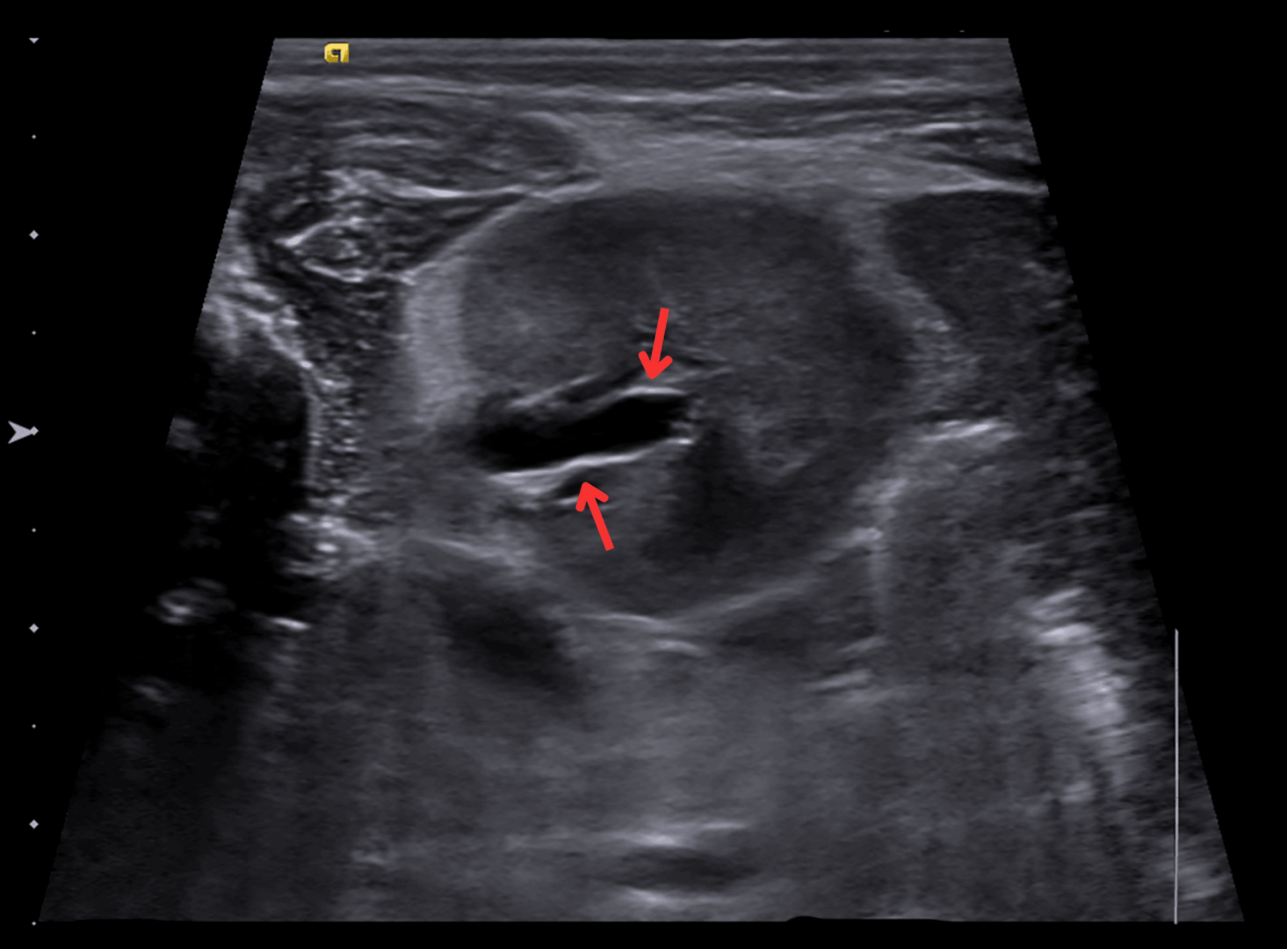
Renal ultrasonography showing urothelial thickening in the infant

Renal ultrasonography obtained on hospital day 2 showed urothelial thickening (Figure 1), and subsequent voiding cystourethrogram (VCUG) was normal, without signs of vesicoureteral reflux or posterior urethral valves. A pediatric nephrologist was consulted, who advised that his urothelial thickening was due to acute changes related to infection and not a sign of congenital urinary tract anomaly. 

While the infant was admitted, the mother herself presented to the emergency department with fatigue and a fever of 101°F. She denied any localizing symptoms; specifically, there was no dysuria, hematuria, urinary frequency, or urinary urgency. Her recent postpartum course had been notable for postpartum hemorrhage related to a retained placenta that required manual extraction. She did not sustain any perineal lacerations as a result of vaginal delivery. She had been breastfeeding her baby without difficulty and denied breast tenderness or erythema. 

On examination, she had a temperature of 100.4º F and a heart rate of 106 beats per minute. There was mild suprapubic tenderness to palpation and no costovertebral angle tenderness. A complete blood count was notable for a white blood cell count of 16.9 K/uL (reference range, 4-11); basic metabolic and liver panels were normal. Urinalysis results are shown in Table 2; urine cultures eventually grew >100,000 colony-forming units per mL of pan-susceptible *E. coli*. 

**Table 2:** Urinalysis results of the mother

Laboratory Result	Result	Reference Value
White blood cells	>150/hpf	0-5/hpf
Leukocyte esterase	3+	Negative
Nitrites	Positive	Negative
Red blood cells	17/hpf	0-4/hpf

**Figure 2:**
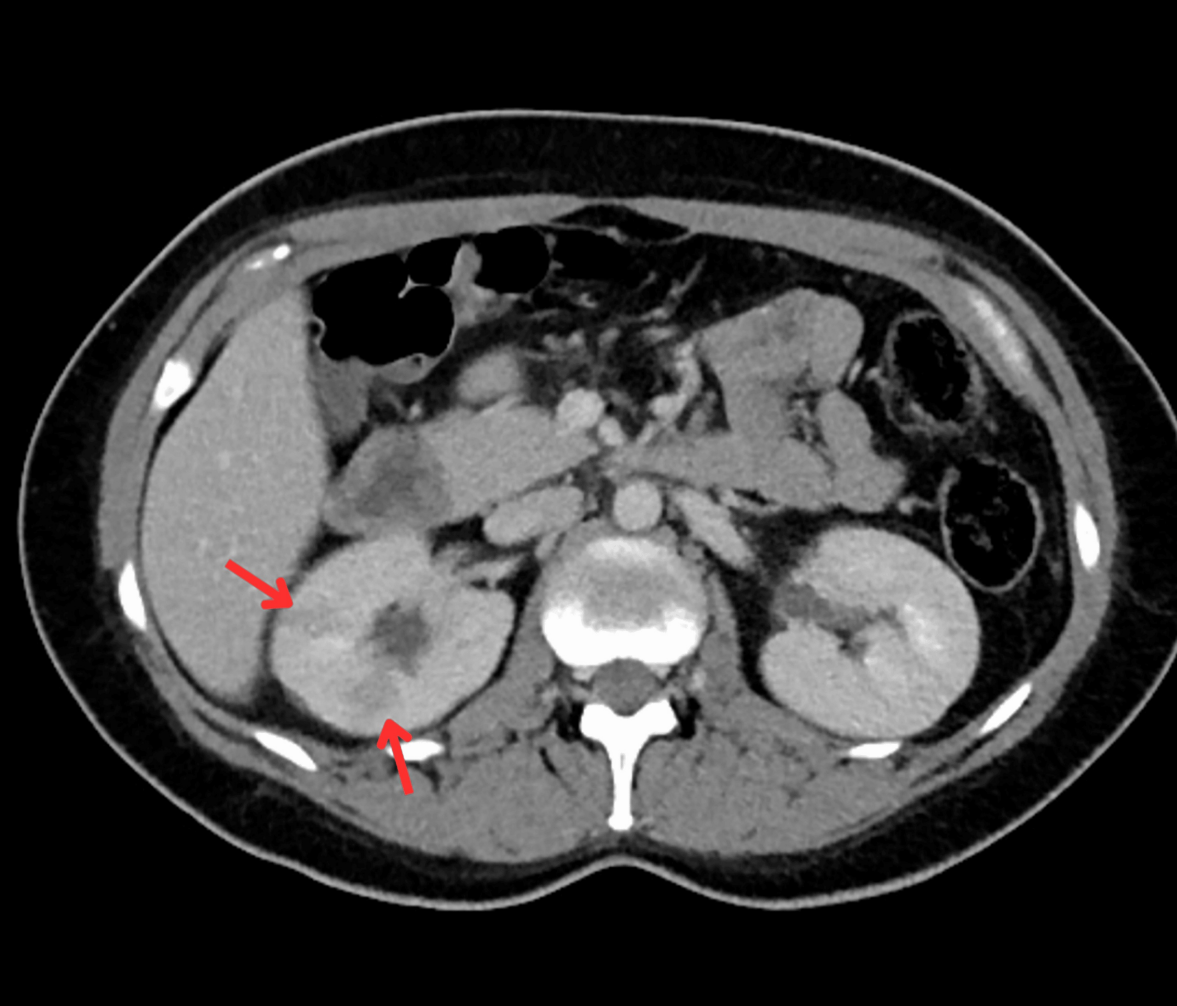
CT imaging of the mother showing hypoenhancement in the right kidney consistent with pyelonephritis

Computed tomography (CT) (Figure 2) of the abdomen and pelvis showed bilateral urothelial thickening and enhancement, as well as right renal parenchymal hypoenhancement (red arrows), consistent with bilateral ascending urinary tract infection with right pyelonephritis. 

Empiric ceftriaxone therapy led to an improvement in her fatigue and fevers, and she was transitioned to oral cephalexin on discharge. 

## Discussion

*E. coli* is a gram-negative bacillus that is most commonly associated with gastrointestinal and genitourinary infections. The bacterium’s adhesive fimbriae allow for attachment to genitourinary tissues, resulting in an inflammatory immune response and subsequent tissue damage [4]. Pyelonephritis most commonly occurs through ascending infection, which may be a result of incomplete bladder emptying or secondary to sexual intercourse; in rare cases, pyelonephritis can be due to hematogenous spread from other sites of infection. Risk factors for pyelonephritis include, but are not limited to, shorter urethral length, diabetes mellitus, extremes of age, sexual activity, and immunosuppression or immune system immaturity [1].  

While patients of all ages and sexes may develop pyelonephritis, pregnant women are considered to be at high-risk, with a prevalence of pyelonephritis as high as 1-2% during the second trimester. In one retrospective cohort study, 2% of women admitted for pyelonephritis were postpartum [5]. Multiple physiologic changes predispose pregnant women to pyelonephritis. First, women have shorter urethrae than men, allowing for more efficient ascending infection. Additionally, during pregnancy, progesterone secretion results in decreased tone in the urinary tract, leading to incomplete bladder emptying [6]. Compression of the urinary bladder or ureters by the growing fetus may also compromise the normal flow of urine [7]. 

Similarly, neonates are predisposed to UTIs, and specifically those caused by *E. coli*, which* *is the most commonly isolated pathogen from neonatal urinary samples, comprising 30-50% of cultured organisms [8-10]. Risk factors for UTIs in neonates include vesicoureteral reflux, posterior urethral valves, and other congenital anomalies of the kidney and urinary tract (CAKUT) [10]. Additionally, uncircumcised male neonates are at increased risk of UTI compared to female and circumcised male neonates, with the foreskin acting as a bacterial reservoir [2,3]. Our infant did not have any CAKUT imaging, but did have uncircumcised status and young age as risk factors. 

It is notable that the mother and her infant in this case were infected by a similarly pan-susceptible E. coli, leading us to hypothesize that it was the same strain of E. coli responsible for both of their infections. This would be supported by data showing that the maternal microbiome plays a role in many neonatal infections. Prenatally, during vaginal delivery, and postnatally with breastfeeding, the fetus is exposed to and acquires portions of the maternal microbiota. One study analyzed mother-infant paired samples of microbiota from several areas of the body and showed that approximately 60% of the fetal microbiome could be attributed to maternal transmission [11]. Another study demonstrated that the maternal gut microbiome at 32 weeks’ gestation and breast milk microbiome predicted the gut microbiome of infants at day of life 14 [12]. Many studies support the role of vertical transmission of infant gut microbiota in utero, with microbes detected on placental specimens [13,14]. Finally, there is specific evidence demonstrating homology between third-trimester maternal vaginal/gastrointestinal and infant respiratory and rectal E. coli strains [15,16]. 

To our knowledge, this is the first documented report of co-occurring pyelonephritis in a mother-infant dyad; however, cases may be underreported. The shared microbiomes between mothers and their infants provide a logical explanation for our patients' presentation. We hypothize that this mother’s gut was populated with pan-susceptible *E. coli* and that the strain was reasonably passed into the infant’s gut during pregnancy or shortly after [12]. Both of these patients then had risk factors for ascending infection, including pregnancy and female sex in the mother and young age with uncircumcised status in the neonate. We acknowledge that because bacterial genome testing was not obtained, we are not able to definitively prove that the two *E. coli* strains in the mother and infant were identical, though it remains plausible based on the mechanisms described. 

## Conclusions

We presented a case involving ascending *E. coli* infection in a mother and her neonate, potentially due to the same *E. coli* strain. Given the large overlap in the microbiome between infants and their mothers, there should be heightened suspicion for shared infections in infants and their mothers in the postpartum period. Pediatricians and internists should ask about maternal infections during history-gathering for pediatric infections and vice versa, as this may reveal otherwise underrecognized connections. 
